# CASS (CyanoAcrylate closure versus Surgical Stripping for incompetent saphenous veins) study: a randomized controlled trial comparing clinical outcomes after cyanoacrylate closure and surgical stripping for the treatment of incompetent saphenous veins

**DOI:** 10.1186/s13063-020-04393-0

**Published:** 2020-06-03

**Authors:** Sungsin Cho, Hyung Sub Park, Taeseung Lee, Seung Jae Byun, Woo-Sung Yun, Shin-Seok Yang, Hyangkyoung Kim, Woo-Shik Kim, Jin Hyun Joh, In Mok Jung

**Affiliations:** 1 Department of Surgery, Kyung Hee University Hospital at Gangdong, Kyung Hee University College of Medicine, Seoul, South Korea; 2 Department of Surgery, Seoul National University Bundang Hospital, Seoul National University College of Medicine, Seongnam, South Korea; 3 Department of Surgery, Wonkwang University Hospital, Wonkwang University School of Medicine, Iksan, South Korea; 4 Department of Surgery, Yeungnam University Medical Center, Yeungnam University School of Medicine, Daegu, South Korea; 5 Department of Surgery, Chung-Ang University Hospital, Chung-Ang School of Medicine, Dongjak-gu, South Korea; 6 Department of Thoracic and Cardiovascular Surgery, National Medical Center, Seoul, South Korea; 7 Department of Surgery, Seoul Metropolitan Government-Seoul National University Boramae Medical Center, Seoul National University College of Medicine, 07061 20 Boramae-ro, Dongjak-gu, Seoul, South Korea

**Keywords:** Varicose vein, Stripping, Endovenous ablation, Occlusion, Quality of life

## Abstract

**Background:**

Several modalities are used for the treatment of varicose veins. Open surgical treatment with ligation and stripping of the saphenous vein has been the standard of care for many years. Endovenous thermal ablation has been shown to be a safe and effective alternative with high, long-term, target-vein closure rates. Despite this, there is the possibility of thermal injury to surrounding structures. The recently introduced cyanoacrylate closure is also considered to be a good alternative and the risk of injury to surrounding structures is minimal. The purpose of this study is to demonstrate the non-inferiority of cyanoacrylate closure with the VenaSeal™ closure system compared to surgical stripping in terms of clinical outcomes for the treatment of incompetent great saphenous veins.

**Methods/design:**

This is an open-label, multicenter, prospective, randomized controlled trial evaluating the non-inferior clinical outcomes of cyanoacrylate closure compared to surgical stripping for the treatment of incompetent saphenous veins. After baseline measurements, participants will be randomly allocated into either the cyanoacrylate closure group or the surgical-stripping group. The primary endpoint of the study is the complete closure rate of the target vein in the cyanoacrylate closure group, and the absence of venous reflux or residual venous tissue after surgical stripping in the surgical-stripping group. These endpoints will be measured by Doppler ultrasound performed by qualified vascular technologists or investigators at 3 months after treatment. Secondary outcomes include perioperative pain, postoperative ecchymosis, clinical assessment (including general and disease-specific quality of life evaluations), complete closure rate, and absence of venous reflux or residual venous tissue at the 12- and 24-month follow-ups, as well as all adverse event rates during the 24-month follow-up period.

**Discussion:**

This multicenter randomized controlled trial is designed to show non-inferiority in terms of complete closure rate of cyanoacrylate compared to surgical stripping for the treatment of incompetent saphenous veins.

**Trial registration:**

Clinical Research Information Service (CRIS), ID: KCT0003203. Registered on 20 September 2018.

## Background

Varicose veins are highly prevalent, and in Western countries, an estimated 23% of adults have varicose veins and 6% have advanced chronic venous disease, including skin changes and healed or active venous ulcers [1]. Although several modalities are used in the treatment of varicose veins, open surgical treatment with ligation and stripping of the saphenous vein, combined with excision of large varicosities, has traditionally been the standard of care for many years. However, surgical stripping is usually associated with a 2- to 3-week recovery period until normal activity can be resumed.

In the 1990s, however, a notable revolution occurred in the treatment of varicose veins, which was introduced by the introduction of minimally invasive endovenous techniques. Endovenous thermal ablation by radiofrequency ablation (RFA) or endovenous laser ablation (EVLA) is a safe and effective alternative with good long-term target-vein closure rates [2]. The results of these endovenous thermal ablations, even in some of the randomized clinical trials compared to previous surgical methods, have consistently been excellent [3, 4]. For this reason, thermal ablation as the first-line treatment of truncal-vein incompetence is now widely accepted and frequently used in clinical practice. However, there is a possibility of thermal injury of the surrounding structures such as nerve injury and skin burns. The use of tumescent anesthesia is a prerequisite in order to prevent thermal injury; however, this is time-consuming and has side effects such as pain, ecchymosis, and hematoma formation [5, 6].

The recently introduced cyanoacrylate closure method is a good alternative since it does not result in thermal injury; thus, there is no need for the use of tumescent anesthesia [7–9], the so-called non-thermal, non-tumescent endovenous technique. Therefore, this method is considered to have more advantages for a patient’s perioperative experience with excellent outcome than conventional surgery. However, to the best of our knowledge, the papers published so far report only the excellent results of the cyanoacrylate closure method alone, a comparison of outcomes with thermal ablation, or a comparison of quality of life only. The purpose and the importance of this study is to demonstrate the non-inferior clinical outcomes of cyanoacrylate closure with the VenaSeal™ closure system compared directly to surgical stripping for the treatment of incompetent saphenous veins. Furthermore, this study could confirm the complete closure rate of cyanoacrylate closure in the long-term period in a multicenter setting, which remains unclear in the long-term.

## Methods/design

### Study objectives, hypothesis, and design

This is an open-label, multicenter, prospective, randomized controlled trial that will evaluate whether the clinical outcomes after cyanoacrylate closure are non-inferior to surgical stripping for the treatment of incompetent saphenous veins (Clinical Research Information Service (CRIS), KCT0003203). After baseline measurements, participants will be randomly allocated into the cyanoacrylate closure group or the surgical-stripping group. Participating centers are in Korea of which Kyung Hee University Hospital at Gangdong, Seoul National University Bundang Hospital, Wonkwang University Hospital, Yeungnam University Medical Center, and Seoul Metropolitan Government-Seoul National University Boramae Medical Center and will participate in patient recruitment and data collection.

### Study population and eligibility

Patients will be eligible for inclusion in the study if they require treatment for incompetent saphenous veins and have one or more of the following symptoms related to the incompetent saphenous vein: aching, throbbing, heaviness, fatigue, pruritus, night cramps, restlessness, discomfort, or swelling. To be eligible for randomization, patients must have identifiable reflux in the great saphenous vein (GSV) for greater than 0.5 s after distal compression and release or Valsalva’s maneuver in the standing or reverse Trendelenburg position. Patients must also have a Clinical, Etiologic, Anatomic, and Pathophysiologic (CEAP) classification score of C2 through C5. Other inclusion and exclusion criteria are listed in Table 1.

**Table 1:** Study eligibility criteria

Inclusion criteria	
Aged between 18 and 80 years	
Reflux in the great saphenous vein of > 0.5 s	
Diameter of the saphenous vein between 2 and 20 mm (standing position)	
One or more of the symptoms related to the incompetent saphenous vein	
CEAP classification of C2 through C5	
Exclusion criteria	
Previous treatment in the targeted vein segment	
Tortuous vein in which the delivery catheter cannot be inserted	
Aneurysm of target-vein segment of > 20 mm	
Daily use of narcotic or pain medications to control pain associated with reflux	
Known hypercoagulable disorder	
Active malignancy	
Regular or current use of systemic anticoagulation	
Previous deep vein thrombosis/pulmonary embolism or active acute superficial thrombophlebitis	
Unable to comply with the schedule and protocol evaluations	
Unable to ambulate	
Unable to provide informed consent	
Currently pregnant or breastfeeding	
Known sensitivity to cyanoacrylate adhesives	
Participation in another clinical study that did not reach the primary endpoint within 30 days prior to enrollment	

*CEAP* Clinical, Etiologic, Anatomic, and Pathophysiologic

### Intervention and assessment schedule

All patients with symptomatic incompetent GSVs will be treated by either cyanoacrylate closure or surgical stripping and followed up for a total of 24 months after treatment. A brief flowchart of the study is presented in Table 2.

**Table 2:** Study protocol items of the trial

	Study period							
	Screening	Procedure	3 days	1 month	3 months	6 months	12 months	24 months
Outpatient visit	√		√	√	√	√	√	√
Physical examination	√		√	√	√	√	√	√
Duplex examination	√		√		√		√	√
Inclusion criteria	√							
**Randomization**		√						
VCSS	√			√	√	√	√	√
Quality of life score – AVVQ	√			√	√	√	√	√
VAS pain score	√	√	√					
Ecchymosis			√	√				
Other adverse events		√	√	√	√	√	√	√

*VCSS* Venous Clinical Severity Score, *AVVQ* Aberdeen Varicose Vein Questionnaire, *VAS* Visual Analog Scale

#### Cyanoacrylate closure with the VenaSeal™ closure system (Medtronic, Minneapolis, MN, USA)

Under all types of anesthesia (general, spinal, regional block, or local anesthesia), the VenaSeal™ closure system is used as per the instructions for use by the manufacturer. In brief, the target vein is accessed and a guidewire is inserted. A 5-Fr introducer sheath/catheter is advanced to the saphenofemoral junction. The catheter tip is positioned 5.0 cm caudal to the junction. With proximal saphenous vein compression using the ultrasound probe, two injections of approximately 0.10 mL cyanoacrylate glue are given 1 cm apart at this location, followed by 3 min of local manual compression. A second single injection is given 3 cm distally after proximal vein compression using the ultrasound probe, and manual compression is performed for 30 s. This latter sequence is repeated throughout the entire length of the target vein to be treated. After completion, the sheath/catheter is removed, and compression is applied to the catheter entry site until hemostasis is achieved. Target-vein occlusion is confirmed by duplex ultrasound.

#### Surgical stripping

All types of anesthesia (general, spinal, regional block, or local anesthesia) can be used. Surgical stripping is performed with a proper incision in the groin, with division and ligation of the saphenous vein and division of all tributaries. The saphenous vein is then removed using a stripper.

#### Adjunctive procedures

Adjunctive procedures, such as phlebectomy of tributaries or sclerotherapy, can be performed at the discretion of the operator.

### Follow-up

Outcomes are recorded at 3 days and 1, 3, 6, 12, and 24 months after treatment (Table 2). The participants should return to the clinic 3 days after treatment and clinical information is collected. This includes the participants’ reporting of perioperative pain using a Visual Analog Scale (VAS); the investigator’s assessment of the presence of ecchymosis, rated using a 0- to 5-point scale (0, none; 1, involving < 25% of the treatment area; 2, 25–50%; 3, 50–75%; 4, 75–100%; 5, extension above or below the treatment segment); and the result of duplex ultrasound. The participants should also return 1 month, 3 months, 6 months, 12 months, and 24 months after treatment in order for clinical information to be collected; this includes the Venous Clinical Severity Score (VCSS) and the evaluation of the participants’ quality of life assessed by the Aberdeen Varicose Vein Questionnaire (AVVQ) and the EuroQoL Five Dimensions Questionnaire (EQ-5D). Duplex ultrasound follow-up is also performed at 3 months, 12 months, and 24 months. Other health problems and all adverse events are monitored at each study visit. The trial follow-up will continue until 24 months after index treatment.

### Outcome measures

The primary endpoint of the study is to evaluate the complete closure of the target vein; this is defined as vein closure along the entire treated vein segment with no discrete segments of patency exceeding 5 cm after cyanoacrylate closure, and the absence of venous reflux or residual venous tissue after surgical stripping. These endpoints will be measured by duplex ultrasound performed by a qualified vascular technologist or investigator at 3 months after treatment. The secondary outcomes include perioperative pain, postoperative ecchymosis, VCSS score, AVVQ, and EQ-5D at each scheduled follow-up visit; all adverse events during the 24-month follow-up period; and the complete closure rate and absence of venous reflux or residual venous tissue at the 12- and 24-month follow-ups.

### Adverse events

Adverse events in clinical trials refer to all adverse events observed in the study subjects and do not necessarily have a causal relationship with treatment. The recording of serious adverse events (SAEs) will conform to the Good Clinical Practice standards and the Research Governance Framework 2005. The analysis of safety-related data will be performed with respect to the frequency of SAEs, including all-cause death, life-threatening events, need for hospitalization, need to extend hospital stay, and permanent significant function impairment. SAEs must be reported by the attending physician to the principal investigator within 24 h after the SAE becomes known.

### Statistical considerations

The purpose of this study is to evaluate the non-inferiority of cyanoacrylate closure to surgical stripping in the treatment of incompetent saphenous veins. A sample size of 73 in each group will achieve 80% power to detect a non-inferiority margin difference between the group proportions of 96% in the cyanoacrylate group and 93% in the surgical-stripping group, assuming a dropout rate of 20%. Randomization will be performed by an independent statistical core at Kyung Hee University Hospital. The allocation of treatment will be done via a web-based randomization system, which returns the treatment group after the input of a participant’s study ID and inclusion/exclusion criteria.

The participants’ characteristics will be summarized using mean and standard deviation for continuous variables and frequency and percentage for categorical variables. A two-sided 95% confidence interval for a difference in occlusion rate between the two treatment groups will be calculated; then, whether the upper limit of confidence interval falls within the pre-determined margin of non-inferiority will be evaluated in order to prove the non-inferiority of cyanoacrylate closure to surgical stripping. Among the secondary outcomes, adverse events will be analyzed using chi-squared tests or Fisher’s exact tests as appropriate. SAEs will be compared using log-rank tests and survival curves constructed using Kaplan-Meier methods.

Significance tests will be two-sided. A *P* value of < 0.05, according to SPSS, will be considered to indicate a statistically significant difference between the groups. SPSS software version 22 (IBM, Armonk, NY, USA) will be used for data analysis.

## Discussion

Since the 1990s, with the advent of minimal invasive modalities for patient treatment having became major issues in medicine, many new modalities have also been developed and introduced into the area of varicose vein treatment.

The turning point in the treatment of varicose veins was the introduction of endovenous treatment techniques. The initial modalities of these endovenous treatments were mainly heat-based treatments, and constantly report anatomical success rates of over 90% [3, 4, 10–12]. In addition to these good treatment results, the greatest advantage of endovenous thermal ablation is that it has less post-procedural pain and fewer related complications, which shortens the time to return to normal activities. However, pain-related factors that benefit from endovenous thermal ablation can be further maximized by the introduction of non-thermal ablation, which is considered the second generation of endovenous treatment.

The aim of the present randomized trial is two-fold. First, it aims to compare anatomical and clinical success at 2 years after cyanoacrylate closure (CAC), with conventional surgery. Several randomized clinical trials have compared CAC with RFA, EVLA, and foam sclerosis but not with conventional surgery [13, 14] since the first report of human use of CAC for the treatment of saphenous-vein incompetent [15]. Therefore, the authors propose the need for a direct comparison of the outcome between CAC and conventional surgery.

Second, the trial aims to demonstrate that CAC is associated with a significant reduction in post-procedural pain. Pain after endovenous thermal ablation is considerable and probably under-reported. Recent reports have reported less pain after RFA than EVLA [16, 17].

According to a recent trial, mechanochemical endovenous ablation reported better results than RFA for pain. Based on these results, non-thermal methods may be expected to have better results in secondary treatment outcomes than thermal [18]. Therefore, we have assumed that CAC could be regarded as a modality to maximize the patient’s comfort in relation to these pains. To further strengthen this hypothesis, the trial was designed using a non-inferiority principle.

In conclusion, the CASS trial is multicenter randomized controlled trial that aims to show a similar anatomical and clinical success rate, with a reduction in post-procedural pain after CAC, compared with conventional surgery.

## Trial status

Recruitment is ongoing with protocol version 1.3 (IRB No. KHNMC 2018–03–030-005). The date of enrollment of the first participant to the trial was 12 April 2018, and the approximate date of completion of recruitment will be 29 February 2020.

## Data Availability

Not applicable

## Abbreviations

RFA: Radiofrequency ablation
EVLA: Endovenous laser ablation
VAS: Visual Analog Scale
AVVQ: Aberdeen Varicose Vein Questionnaire
EQ-5D: EuroQoL Five Dimensions Questionnaire
VCSS: Venous Clinical Severity Score
CEAP classification: Clinical, Etiologic, Anatomic, and Pathophysiologic classification
SAEs: Serious adverse events
